# P-955. Expansion of antimicrobial stewardship (AMSP) and infection control program (ICP) of ICMR in secondary care hospitals

**DOI:** 10.1093/ofid/ofaf695.1157

**Published:** 2026-01-11

**Authors:** Arya S Kumar, Radhika T. K, Sanjeev Singh

**Affiliations:** Department of Infection control & Epidemiology, kochi, Kerala, India; Department of Infection control & Epidemiology, kochi, Kerala, India; Department of Infection control & Epidemiology, kochi, Kerala, India

## Abstract

**Background:**

Antimicrobial resistance (AMR) is a growing global health challenge, particularly in healthcare settings where antibiotics are often misused. Antimicrobial stewardship programs (AMSP) and infection control practices (ICP) are crucial for combating AMR. While tertiary care hospitals have successfully adopted these programs, secondary care hospitals face significant obstacles, such as limited resources and lack of awareness.

**Figure:**
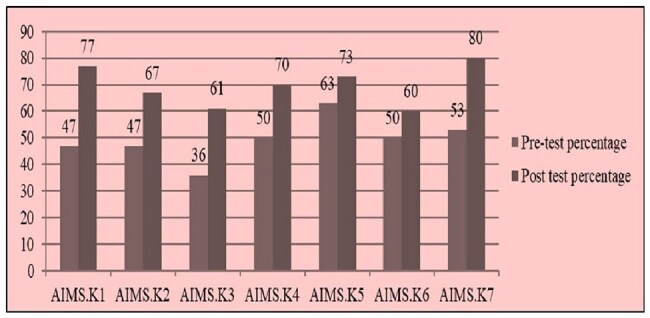
IPC pre and post test percentage assessments

**Figure:**
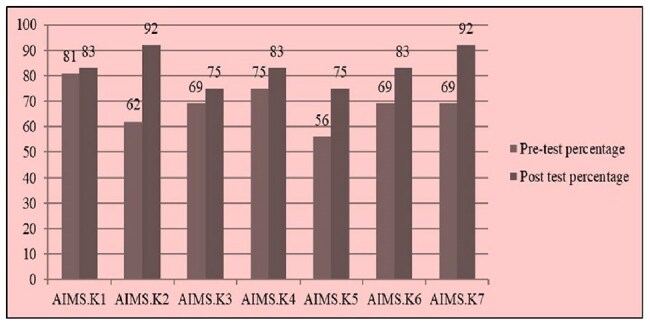
AMSP pre and post test assessments

**Methods:**

This was a quasi-experimental, multicenter study conducted from August 2021 to August 2023 and mentored by Amrita Institute of Medical Sciences, Kochi, Kerala in collaboration with Indian Council of Medical Research and Pfizer. It involved seven hospitals—four mid-level and three small-level facilities. The project was divided into three phases: pre-implementation, implementation, and post-implementation. Baseline assessments of antibiotic use and healthcare workers' knowledge were conducted. AMSP and ICP committees were formed in each hospital, and hybrid training sessions were organized for staff. Antibiotic prescription audits and infection control measures were monitored throughout the study, and post-implementation data were collected for analysis.

**Results:**

The results showed improved antibiotic prescribing and infection control practices in the participating hospitals. Full AMSP implementation was achieved in two hospitals, with the others facing challenges such as staff shortages and limited resources. However, knowledge and practices improved in all hospitals, with notable reductions in antibiotic misuse and HAIs

**Conclusion:**

The study demonstrated the feasibility of implementing AMSP and ICP in secondary care hospitals despite challenges. Continued advocacy, training, and resource support are essential for sustaining these improvements and reducing AMR in India's healthcare system.

**Disclosures:**

All Authors: No reported disclosures

